# A Hybrid Unsupervised Approach for Retinal Vessel Segmentation

**DOI:** 10.1155/2020/8365783

**Published:** 2020-12-10

**Authors:** Khan Bahadar Khan, Muhammad Shahbaz Siddique, Muhammad Ahmad, Manuel Mazzara

**Affiliations:** ^1^Department of Telecommunication Engineering, Faculty of Engineering, The Islamia University of Bahawalpur, Bahawalpur, Pakistan; ^2^Department of Electronic Engineering, Faculty of Engineering, The Islamia University of Bahawalpur, Bahawalpur, Pakistan; ^3^Department of Computer Science, National University of Computer and Emerging Sciences, Islamabad, Chiniot-Faisalabad Campus, Chiniot 35400, Pakistan; ^4^Institute of Software Development and Engineering, Innopolis University, Innopolis, Russia

## Abstract

Retinal vessel segmentation (RVS) is a significant source of useful information for monitoring, identification, initial medication, and surgical development of ophthalmic disorders. Most common disorders, i.e., stroke, diabetic retinopathy (DR), and cardiac diseases, often change the normal structure of the retinal vascular network. A lot of research has been committed to building an automatic RVS system. But, it is still an open issue. In this article, a framework is recommended for RVS with fast execution and competing outcomes. An initial binary image is obtained by the application of the MISODATA on the preprocessed image. For vessel structure enhancement, B-COSFIRE filters are utilized along with thresholding to obtain another binary image. These two binary images are combined by logical AND-type operation. Then, it is fused with the enhanced image of B-COSFIRE filters followed by thresholding to obtain the vessel location map (VLM). The methodology is verified on four different datasets: DRIVE, STARE, HRF, and CHASE_DB1, which are publicly accessible for benchmarking and validation. The obtained results are compared with the existing competing methods.

## 1. Introduction

The most essential sensory system for gathering information, navigation, and learning is the human visual system [1]. The retina is the sensitive part of the eye that contains fovea, light receptors, Optic disk, and macula. The retina is a layered tissue, coating the interior of the eye, which is an initial sensor of the communication system and gives a sense of sight. Moreover, it allows understanding the colors, dimensions, and shape of objects by processing the amount of light it reflects or emits. Retina image of an eye is captured with a fundus camera [2]. RGB photographs of the fundus are the protrusion of the internal surface of an eye. Imaging of the retina has emerged swiftly and now one of the most common practices in healthcare and for screening the patients suffering from ophthalmologic or systemic diseases. For identify ing numerous ophthalmologic diseases, the ophthalmologist uses vessel condition as an indicator which is a vital component in retinal fundus images.

Critical diagnostic to eye diseases in human retinal images can be indicated by its shape analysis, its appearance, blood vessels, morphological features, and tortuosity [3]. Structure of RVS is also used for screening of brain and heart stock diseases [4, 5]. Retinal vessel structures play a significant role among other structures in fundus images. RVS is the elementary phase utilized for the examination of retina images [6]. Vascular-related diseases are diagnosed with the help of vessel delineation which is an important component of medical image processing. Additionally, ongoing research in the area of deep learning suggested multiple approaches with emphasis on the separation and the delineation of the vasculature.

**Figure 1:**
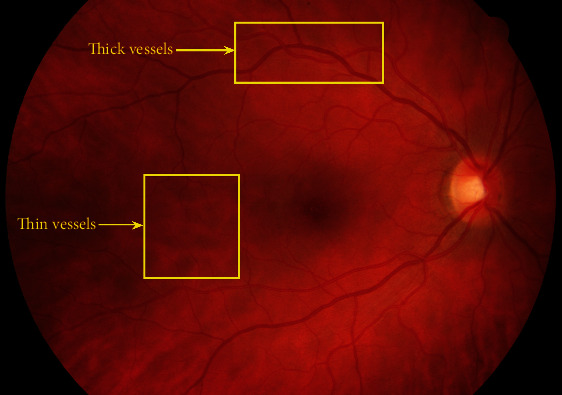
Graphical representation of thinner and thicker vessels.

The inadequate number of images and having low-contrast in publicly available retina datasets is challenging for deep learning-based research. A dataset having a large number of retina images captured with a different imaging system and under diverse environmental conditions is required to train the supervised network. Deep learning-based methods will aid to control blindness, timely and precise identification of diseases for successful remedy, and thus vividly increase the life quality of patients with eye ailments [7]. RVS is a very difficult task due to many reasons:

1. The structure and formation of retinal vessels are very complex and there is a prominent dissimilarity in various local parts regarding the shape, size, and intensity in vessels.
2. Some structures have the same intensity and shape as vessels, e.g., hemorrhage. Moreover, there are also thin microvessels, whose width is normally between ranges from one to a few pixels and which can be easily mixed with the background. There are irregular illumination in the images and having low-varying contrast [7, 8]. Typically, noise in fundus images is added by the image-capturing procedure such as artifact on the lens or movement of the patient [9]. It is hard to differentiate vessels from other structures that are similar or noises in the retina image. In other words, thicker vessels are more prominent in comparison to the thinner ones as shown in Figure 1

(3) Different manual graders have different segmentation results. Manual RVS is also a very hard and tedious task. Over the recent two decades, automatic RVS has caught noteworthy attention and numerous such techniques are developed but they have performance degradation with the change of datasets. Some of the techniques are not fully automatic while others are incapable to handle pathological images. Some of these methods are evaluated on the datasets having a limited number of images while others have problems of oversegmentation or undersegmentation with abnormal images [10]. Hence, the dilemma of perfect RVS is still not answered.

Automated RVS techniques provide incredible support to the ophthalmologist in terms of identification and medication of numerous ophthalmological abnormalities. In this article, an automatic unsupervised approach is developed for RVS that consists a combination of the preprocessing steps, segmentation, vessel structure-based enhancement, and postprocessing steps. The preprocessing steps aim at exterminating noise and improving the contrast of the fundus image. Segmentation is performed by using the Modified Iterative Self Organizing Data Analysis Technique (MISODATA) to acquire a binary image that is fused with the segmented image of the Combination Of Shifted Filter Responses (B-COSFIRE). Then, the fused image is multiplied with the enhanced image of the B-COSFIRE to obtain the initial vessel location map (VLM). Lastly, the VLM and the fused image are combined by logical OR-type operators to obtain final results. In a nutshell, the main contributions of this research are the following:

1. A mask image is not provided with all retina datasets. Automatic masking creation is proposed for each image to extract ROI which suppresses the false positive rate (FPR).
2. The proposed efficient denoising process (preprocessing steps) improves the selection of a suitable threshold.
3. The basic ISODATA algorithm only one-time process the retina image locally and then globally, which sometimes makes it unable to find an optimal threshold. The modified ISODATA technique is introduced to find the global threshold of the entire image which is compared and equated with the individual local threshold of each segment in order to find the optimal threshold for more precise detection of vessels.
4. The vessel location map (VLM) is a new scheme to achieve better performance. In this scheme, the background noise eradication and vessel enhancement are accomplished independently.
5. A distinctive postprocessing steps (AND-type and OR-type operations) to reject misclassified foreground pixels.

## 2. Related Works

Numerous methodologies for RVS have been developed in literature [4, 10]. These methodologies are arranged into two sets: supervised and unsupervised procedures. Supervised techniques utilizing a trained classifier for pixel classification into the foreground or background. Supervised techniques utilized various classifiers, for instance, adaptive boosting (AdaBoost), support vector machines (SVM), neural networks (NN), Gaussian mixture models (GMM) and *k*-nearest neighbors (*k*-NN).

A RVS method utilizing a supervised *k*-NN classifier for isolation of foreground and background pixels was recommended by Niemeijer et al. [11], with a feature vector (FV) formation based on a multiscale (MS) Gaussian filter. Staal et al. [12] projected an equivalent RVS methodology using an FV generated based on a ridge detector. A feed-forward NN built classifier was applied by Marin et al. [13], using 7-D FV generated based on moment-invariant.

An SVM-based approach was presented by Ricci et al. [14], utilizing FV constructed through a rotation-invariant linear operator and pixel intensity. An AdaBoost classifier was suggested by Lupascu et al. [15], utilizing a 41 − *D* feature set. An ensemble-based RVS system applying a simple linear iterative clustering (SLIC) algorithm was presented by Wang et al. [16]. A GMM classifier-based scheme was recommended by Roychowdhury et al. [17], utilizing 8 − *D* FV extracted from the pixel neighborhood on first and second-order gradient images.

Zhu et al. [18] offered an extreme learning machine(ELM) based RVS scheme utilizing a 39 − *D* FV generated by morphological and local attributes combined with attributes extracted from phase congruency, Hessian, and divergence of vector fields (DVF). Tang et al. [19] recommended an SVM-based RVS scheme utilizing an FV created based on MS vessel filtering and the Gabor wavelet features. A random forest classifier-based RVS system was proposed by Aslani et al. [20], utilizing a 17 − *D* FV created based on MS and the multiorientation Gabor filter responses and intensity feature combined with feature extracted from vesselness measure and B-COSFIRE filter.

A directionally sensitive vessel enhancement-based scheme combined with NN derived from the U-Net model was presented in [21]. Thangaraj et al. [22] constructed a 13 − *D* FV from the Gabor filter responses, Frangi's vesselness measure (1*D*), local binary pattern feature (1*D*), Hu moment invariants (7*D*), and grey-level cooccurrence matrix features (3*D*) for RVS utilizing NN-based approach. Memari et al. [23] recommended an arrangement of various enhancement techniques with the AdaBoost classifier to segregate foreground and background pixels.

A three-stage (thick vessel extraction, thin vessel extraction, and vessel fusion-based) deep learning approach were proposed in [24]. Guo et al. [25] suggested an MS deeply supervised network with short connections (BTS-DSN) for RVS. Local intensities, local binary patterns, a histogram of gradients, DVF, higher-order local autocorrelations, and morphological transformation features were used for RVS in [26]. Random forests were used for the selection of feature sets which were utilized in combination with the hierarchical classification methodology to extract the vessels.

Alternatively, unsupervised systems are categorized based on matched filtering (MF), mathematical morphology (MM), and multiscale-based approaches. In matched filtering approaches, thick and thin vessels are extracted by the selection of large and small filter kernels, respectively. However, the application of large kernels can accurately detect major vessels with the misclassification of thin vessels by increasing its width. Similarly, smaller kernels can accurately extract thin vessels along with the extraction of thick vessels in reduced widths. To obtain a complete vascular network, a conventional MF technique can be applied with a large number of diverse filter masks in various directions.

Similar methods were employed using MF [27–32], combined filters [33], COSFIRE filters [3, 5, 34–36], Gaussian filters [37], wavelet filters [38], and Frangi's filter [39]. The MM-based approaches are utilized for isolating retinal image segments such as optic disk, macula, fovea, and vasculature. Morphological operators utilized the application of structuring elements (SE) to images for extraction and representation of region contours. A morphological operation for detecting particular structures has the benefit of speed and noise elimination. But they are unable to achieve the known vessel cross-sectional shape. Moreover, there is an issue to extract extremely tortuous vessels in case of superimposing large SE. Morphological operations were utilized for both enhancement and RVS [2, 40–44]. On the other hand, retinal blood vessels of variable thickness at various scales were obtained by multiscale approaches [45–50].

## 3. Proposed Model

The complete structure of the proposed RVS framework is introduced in this section. The information and description of every stage are also presented in subsections.

### 3.1. Overview

**Figure 2:**
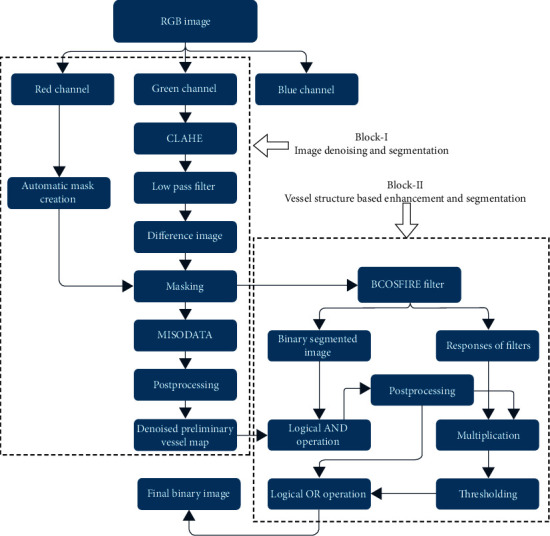
Sequential sketch of the proposed framework.

The proposed framework consists of two major blocks to obtain a final binary image: (1) retina image denoising and segmentation and (2) vessel structure-based enhancement and segmentation. The key objective of this framework is to extract vasculature excellently along with the elimination of noise and supplementary disease falsifications. The complete structure of the proposed framework is labeled in Figure 2. In which Block-I consists of the selection of suitable retina channel, contrast enhancement, noise filtering, region of interest (ROI) extraction, thresholding, and post processing steps. Block-II includes the application of B-COSFIRE filter, logical operations, and postprocessing steps. The initial binary vessel map of Block-I is fused with the B-COSFIRE filter segmented image in Block-II. Then, it is multiplied with the B-COSFIRE filter-enhanced image which is further thresholded. This output image is combined with the initial postprocessed image by the logical OR-type operation to obtain the final binary.

### 3.2. Block-I: Retina Image Denoising and Segmentation

In the first block, the retina image is passed through selected techniques to extract the initial denoised vessel map. The green band of the RGB retina image is extracted and nominated for subsequent operation due to its noticeable contrast difference between the vessel and other retina structures. The RGB retina images generally have contrast variations, low resolution and noise. To avoid such variations and produce more appropriate image for further processing, the vessel light reflex elimination and background uniformity operations are performed. Retinal vessel structures have poor reflectance when equated to other retinal planes. Some vessels contain a bright stripe (light reflex) which runs down the central length of the vessel. To overcome this problem, a disc-shape opening operator with a 3-pixel width SE is used on the green plane. A minimal value of disc width is selected to avoid the absorption of close vessels. The background uniformity and smoothness of random salt-and-pepper noise are obtained by the application of a 3 × 3 mean filter. Additional noise flattening is achieved with the application of a Gaussian kernel of size 9 × 9, mean = 0, and variance 1.8.

**Figure 3:**
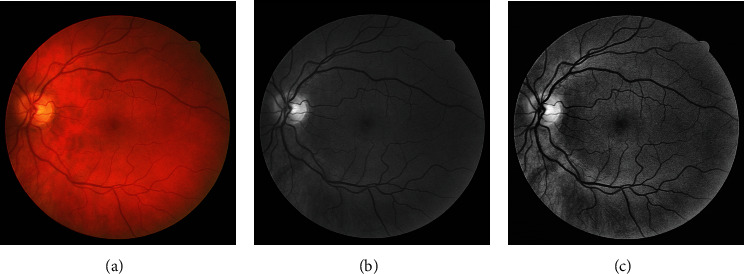
(a) DRIVE dataset color image. (b) Green plane. (c) CLAHE output.

**Figure 4:**
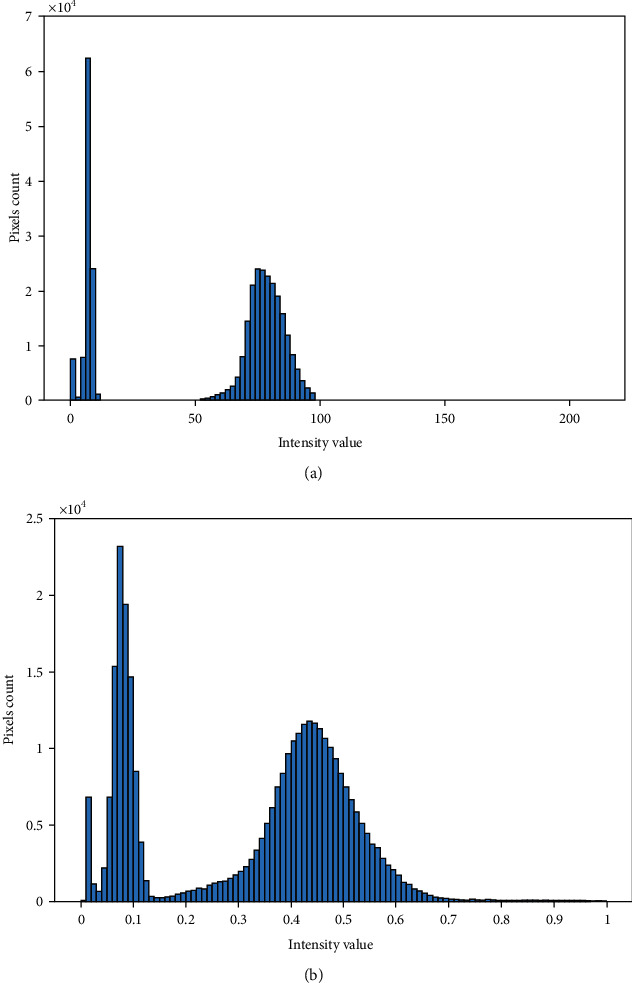
Histogram pictorial effects. (a) Green plane. (b) CLAHE.

CLAHE [51, 52] is applied on the preprocessed green channel to make vessel structures prominent. The CLAHE operation divides the input image into blocks (size 8 × 8 in our case) with the constraint of contrast improvement which is set to 0.01. The clip limit suppresses the noise level and escalates the contrast. The effect of the CLAHE process (*I*_clahe_) along with the green plane is displayed in Figure 3. Histogram-based graphical demonstration of the contrast improvement operations is displayed in Figure 4. An averaging filter of size 49 × 49 is applied for smoothness and elimination of anatomical regions (e.g., optic disk, macula, and fovea). *I*_avg_ symbolizes the output image of the averaging filter. The difference image (*I*_d_) is computed for all pixels as follows.

(1) $$\begin{array}{c} I_{\text{d}}\left(m,n\right)=I_{\text{avg}}\left(m,n\right)-I_{\text{clahe}}\left(m,n\right). \end{array}$$

The extra regions of the retinal image are cropped by the utilization of the masking method to extract ROI which reduced the computational complexity. An automatic mask is created from the red band of the retinal image. The reason behind using the red channel for mask construction is that it has a good vessel-background dissimilarity. The automatic mask is created for all datasets because the mask image is not available in some datasets. *I*_d_ is thresholded by the MISODATA algorithm. The subsequent procedure is used to compute the threshold level, and the application of MISODATA is shown in Algorithm 1.

**Figure 5:**
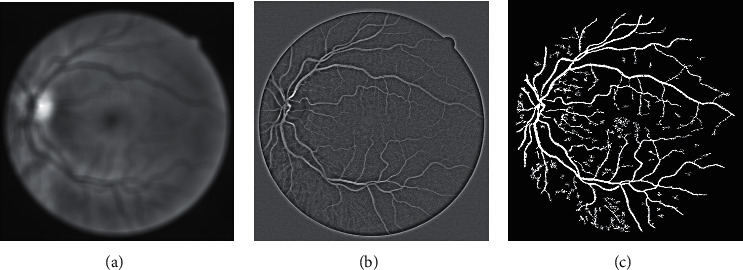
(a) Average filter image. (b) Difference image. (c) Segmented initial vessel map.

The isolated pixels with an area less than 25 pixels in the image (*I*_*s*_1__) are trimmed and fused with the B-COSFIRE filter segmented image of Block-II by AND-type operation. The physical stats (eccentricity and area) are utilized for the rejection of nonvessel structures. The vessel structures have a higher area and eccentricity as their pixels are linked and having an elongated structure.  Figure 5 indicates the graphical results of the *I*_avg_, *I*_d_, and *I*_*s*_1__.

### 3.3. Block-II: Vessel Structure-Based Enhancement and Segmentation

**Figure 6:**
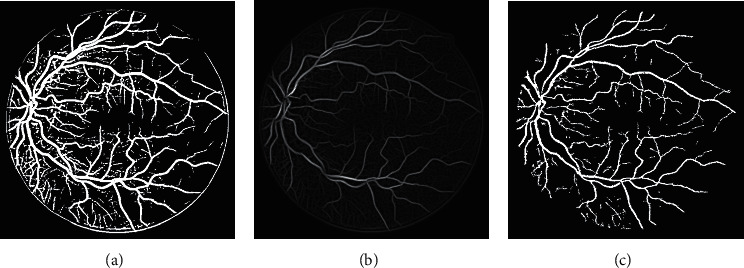
(a) B-COSFIRE binary segmented image (b) Enhanced image based on symmetric-asymmetric filter responses. (c) AND-type operation output.

**Figure 7:**
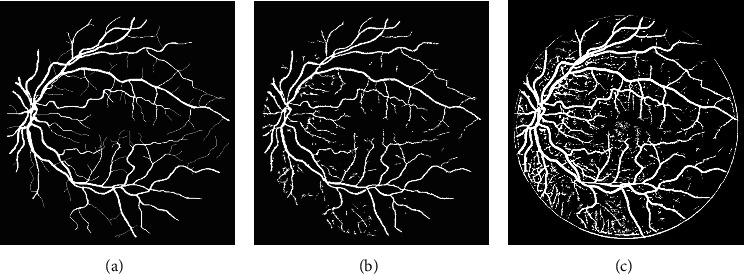
Inspecting the results of AND-type operator. (a) Manual image. (b) AND-type. (c) OR-type.

**Figure 8:**
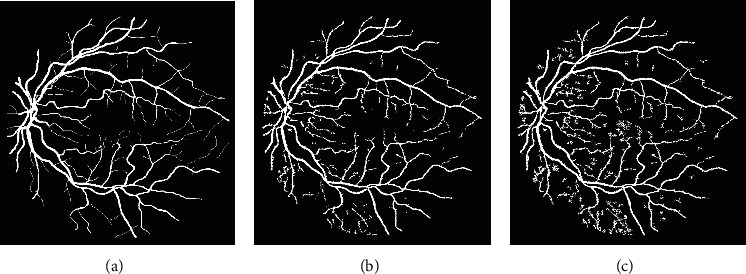
Inspecting the results of AND-type operator. (a) Manual image. (b) AND-type. (c) without AND-type.

**Figure 9:**
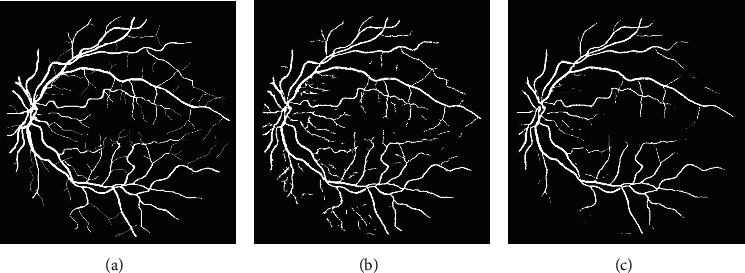
Inspecting the results of OR-type operator. (a) Manual image. (b) OR-type. (c) without OR-type.

In Block-II, the masked image of the Block-I is used as an input for vessel structure-based enhancement and RVS. B-COSFIRE filter [5] is applied for contrast improvement of vessel structures that will enhance noise also along with the enhancement of vessel structures if the image is not preprocessed. Therefore, the masked image is used for further processing. B-COSFIRE filter produced two results: binary segmented image (*I*_*s*_*C*__) and vessel structure-based enhanced image (*I*_*E*_*C*__). The outputs of B-COSFIRE filter are displayed in Figure 6. The AND-type operation is used to combine *I*_*s*_1__ with *I*_*s*_*C*__ that produced output image denoted by *I*_And_. The effect of AND-type operation is shown in Figure 7, which demonstrates that if an alternative operator like OR-type is utilized, it will introduce noise and misclassification. The advantage of using an AND-type operator is exposed in Figure 8 by displaying the visual results with and without using the AND-type operator. The *I*_AND_ is postprocessed (*I*_*p*_1__) and multiplied with *I*_*E*_*C*__ which is further thresholded to obtain a segmented image (*I*_*s*_2__). Pixel-by-pixel multiplication aims at ensuring the detection of vessels at their correct position. The logical OR-type operation is used to produce the final result by coupling of *I*_*p*_1__ and *I*_*s*_2__. The visual effects of the OR-type operator are presented in Figure 9.

The B-COSFIRE filter application includes convolution with difference of Gaussian (DoG) filters, its blurring effects, shifting the blurred responses, and an approximate point-wise weighted geometric mean (GM). A DoG function DoG_*σ*_(*x*, *y*) is given by [5]

(2) $$\begin{array}{c} {\text{DoG}}_{\sigma }\left(x,y\right)=\frac{1}{2{\pi \sigma }^{2}}\exp\left(-\frac{x^{2}+y^{2}}{2\sigma^{2}}\right)-\frac{1}{2\pi {\left(0.5\sigma \right)}^{2}}\exp\left(-\frac{x^{2}+y^{2}}{2{\left(0.5\sigma \right)}^{2}}\right), \end{array}$$

where *σ* is the standard deviation (SD) of the Gaussian function (GF) that decides the range of the boundary. 0.5*σ* is manually set SD value of the internal GF, and (*x*, *y*) symbolizes the pixel position of the image. Response of DoG filter *C*_*σ*_(*x*, *y*) with kernal function of DoG_*σ*_(x − x′, y − y′) has been estimated by convolution, where (*x*′, *y*′) denotes pixels intensity distribution.

(3) $$\begin{array}{c} C_{\sigma }\left(x,y\right)={\left|I*\text{Do}{\text{G}}_{\sigma }\right|}^{+}, \end{array}$$

where |^.^|^+^ represents the half-wave rectification process to reject negative values.

**Figure 10:**
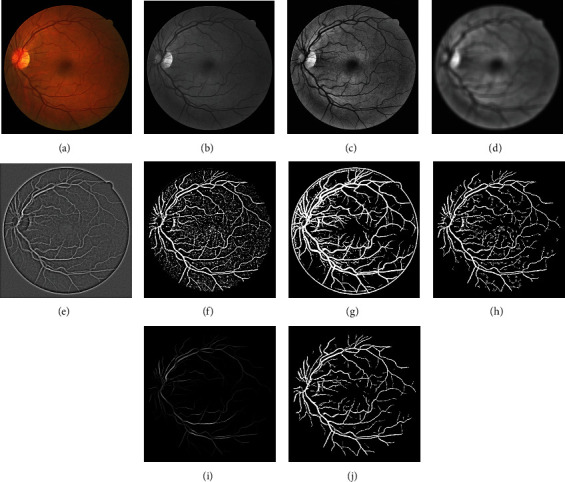
Step-by-step visual results of the proposed method using image from the DRIVE dataset. (a) Color input image. (b) Green plane. (c) CLAHE. (d) Low-pass filter. (e) Difference output. (f) MISODATA. (g) Binarized vessel map of B-COSFIRE. (h) AND operator [*f*, *g*]. (i) B-COSFIRE enhanced image. (j) Final result.

In the B-COSFIRE filter, three factors (*σ*_*i*_, *ρ*_*i*_, ∅_*i*_) are used to represent each point *i*, where *σ*_*i*_ = SD of the DoG filter, while *ρ*_*i*_ and ∅_*i*_ denote the polar coordinates. This set of parameters is indicated by S = {(*σ*_*i*_, *ρ*_*i*_, ∅_*i*_) | *i* = 1, ⋯, *n*}, where *n* represents the figure of measured DoG responses. The blurring process indicates the calculation of the extreme limit of the weighted thresholded responses of a DoG filter. The blurring operation is shown as follows.

(4) $$\begin{array}{c} \sigma^{\prime }=\sigma_{0}^{\prime }+\alpha \rho_{i}, \end{array}$$

where *σ*_0_′ and *α* are constants. Each DoG-blurred outcome is moved in the reverse direction to ∅_*i*_ by a gap *ρ*_*i*_, and as a result, they can merge at the support center of the B-COSFIRE filter. Blurred and shifted responses of the DoG filter is indicated by *S*_*σ*_*i*_,*ρ*_*i*_,∅_*i*__(*x*, *y*) for every tuple (*σ*_*i*_, *ρ*_*i*_, ∅_*i*_) in set *S*. The *i*_th_ blurred and shifted response of the DoG filter is defined as

(5) $$\begin{array}{c} S_{\sigma_{i},\rho_{i},\emptyset_{i}}\left(x,y\right)=\max_{x^{\prime },y^{\prime }}\left\{c_{\sigma_{i}}\left(x-\delta x_{i}-x^{\prime },y-\delta y_{i}-y^{\prime }\right)G_{\sigma_{i}}\left(x^{\prime },y^{\prime }\right)\right\}, \end{array}$$

where −3*σ*′ ≤ *x*′, *y*′ ≤ 3*σ*′. The output of the filter is shown as GM of all the blurred and shifted DoG responses.

(6) $$\begin{array}{c} r_{S}\left(x,y\right)={\left|\left(\overset{\left|S\right|}{\underset{t=1}{\prod }}\;{\left({\left(S_{\sigma_{i},\rho_{i},\emptyset_{i}}\left(x,y\right)\right)}^{\omega_{i}}\right)}^{\overset{\left|S\right|}{\underset{i=1}{\sum }}\;\omega_{i}}\right)\right|}_{t}, \end{array}$$

where *ω*_*i*_ = exp^−*ρ*_*i*_^2^/2*σ*^2^^ and |^.^|_*t*_ symbolizes the thresholding response at *t*, (0 ≤ *t* ≤ 1). Equation (6) represents the AND-type operation that is attained by the B-COSFIRE filter only when all DoG filter responses *S*_*σ*_*i*_,*ρ*_*i*_,∅_*i*__ are larger than zero. The overall step-by-step visual results according to the block diagram (Figure 2) are portrayed in Figure 10.

## 4. Experimental Outcomes and Deliberation

This section will provide the information about datasets, performance metrics, analysis of experimental results, and time complexity of the proposed method.

### 4.1. Datasets

The proposed system obtained remarkable results on the freely online available datasets: DRIVE [11, 12], STARE [53], HRF [54], and CHASE_DB1 [55]. The magnificence of the framework is justified in terms of assessment with state-of-the-art systems. The datasets used for endorsement of the suggested framework are encapsulated in Table 1. The manually labeled results in all datasets are utilized as a gold standard for performance assessment of the proposed framework.

### 4.2. Performance Judgment Parameters

The quantitative results are obtained by equating the proposed segmentation's with the manual segmentation available on each dataset. There are numerous performance standards mentioned in the literature. The performance metrics used for evaluation of the proposed framework are visible in Table 2. Six performance standards (Acc, Sn, Sp, AUC, MCC, and CAL) are selected for the justification of the proposed methodology. The Acc metric tells about the overall valuation of the proposed method. Sn is a measure of the quantity of correctly classified vessel pixels, while Sp is an assessment of the competency of differentiating nonvessel pixels. The AUC is the ratio of Sn and Sp. The MCC [5, 56] is a more appropriate indicator of the accuracy of binary categorization in the case of unbalanced structures. For a comprehensive judgment of the superiority of segmentation, the CAL metric [57, 58] is computed. This metric provides justification based on the properties (connectivity-area-length) of the segmented structures beyond the correctly classified image pixels.

In Table 2, *N* = TN + TP + FN + FP, *S* = (TP + FN)/*N* and *P* = (TP + FP)/*N* [58]. The terms TP, TN, FP, and FN denote the true positive (exactly matched vessel pixels), true negative (exactly matched nonvessel pixels), false positive (invalidly predicated vessel pixels), and false negative (invalidly predicated nonvessel pixels), correspondingly.

Let *I*_*S*_ be the extracted final binary image and *I*_*G*_ the corresponding manual segmented image. The considered metric evaluates the following [57, 58]:

- Connectivity (C): it calculates the fragmentation grade of *I*_*S*_ with respect to the manual segmentation *I*_*G*_ and penalizes fragmented segmentation. It is computed as

(7) $$\begin{array}{c} C\left(I_{S},I_{G}\right)=1-\min\left(1,\frac{\left|{\#}_{C}\left(I_{G}\right)-{\#}_{C}\left(I_{S}\right)\right|}{\#\left(I_{G}\right)}\right), \end{array}$$

where #_*C*_(·) sums the linked segments while #(·) measures the number of vessel pixels in the considered binary image

(ii) Area (A): it estimates the intersecting area between *I*_*S*_ and *I*_*G*_, based on the Jaccard coefficient. Let *δ*_*ε*_(·) be a morphological dilation that utilizes a disc structuring element (SE) with a radius of *ε* pixels. The magnitude *A* is calculated as follows:

(8) $$\begin{array}{c} A\left(I_{S},I_{G}\right)=\frac{\#\left(\left(\delta_{\varepsilon }\left(I_{S}\right)\cap \left(I_{S}\cap I_{G}\right)\cup \left(I_{S}\cap \delta_{\varepsilon }\left(I_{G}\right)\right)\right)\right)}{\#\left(I_{G}\cup I_{S}\right)}. \end{array}$$

The value of *ε* controls the tolerance to lines of various sizes. We set *ε* = 2

(iii) Length (L): it determines the equivalent degree between *I*_*S*_ and *I*_*G*_ by computing the length of the two line networks:

(9) $$\begin{array}{c} L\left(I_{S},I_{G}\right)=\frac{\#\left(\left(\varphi \left(I_{S}\right)\cap \delta_{\beta }\left(I_{G}\right)\right)\cup \left(\delta_{\beta }\left(I_{S}\right)\cap \varphi \left(I_{G}\right)\right)\right)}{\#\left(\varphi \left(I_{S}\right)\cup \varphi \left(I_{G}\right)\right)}, \end{array}$$

where *φ*(·) is a skeletonization process and *δ*_*β*_(·) is a morphological dilation with a disc SE of *β* pixel radius. The value of *β* controls the tolerance to dissimilarity of the line tracing output. We set *β* = 2. The final assessment parameter, named CAL, is demarcated as *f*(*C*, *A*, *L*) = *C* · *A* · *AL*.

### 4.3. Experimental Results and Inspection

**Figure 11:**
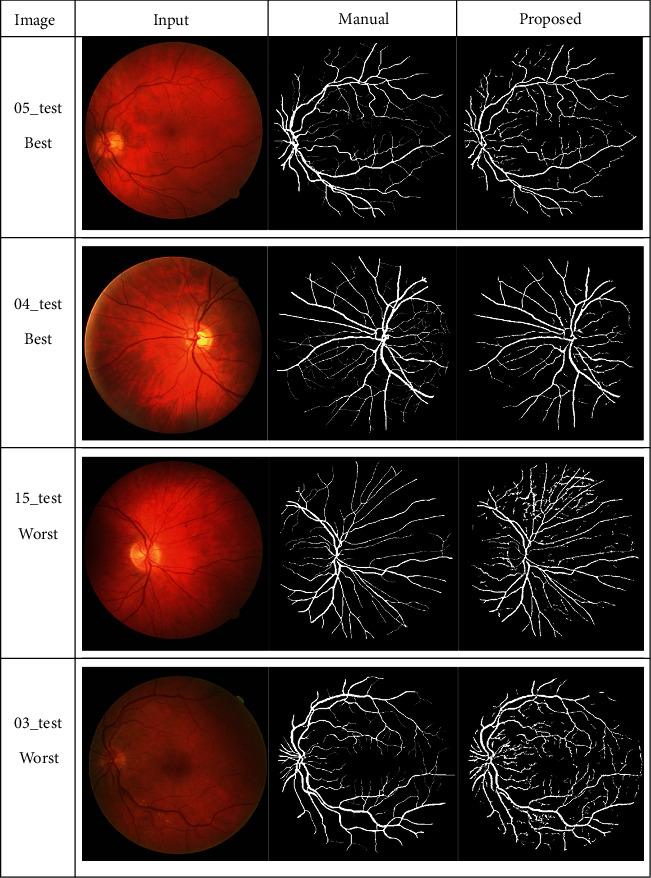
Visual effects of the best and worst cases from the DRIVE dataset.

**Figure 12:**
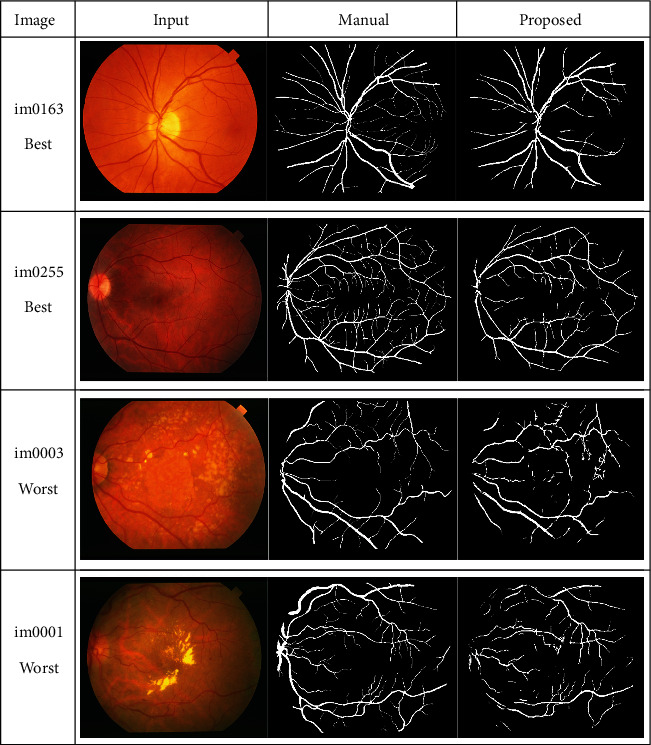
Visual effects of the best and worst cases from the STARE dataset.

**Figure 13:**
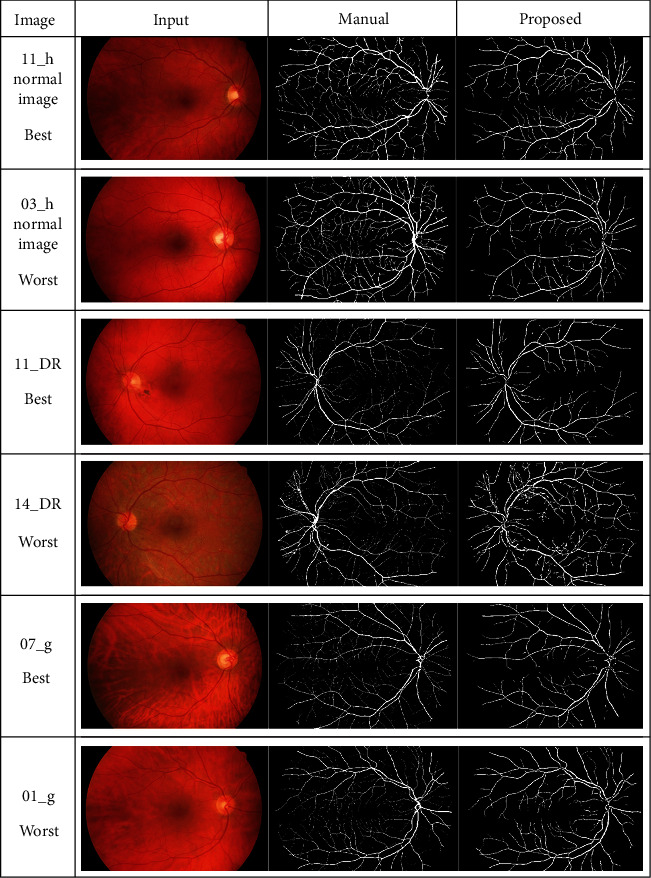
Visual results of the best and worst cases from the HRF dataset.

**Figure 14:**
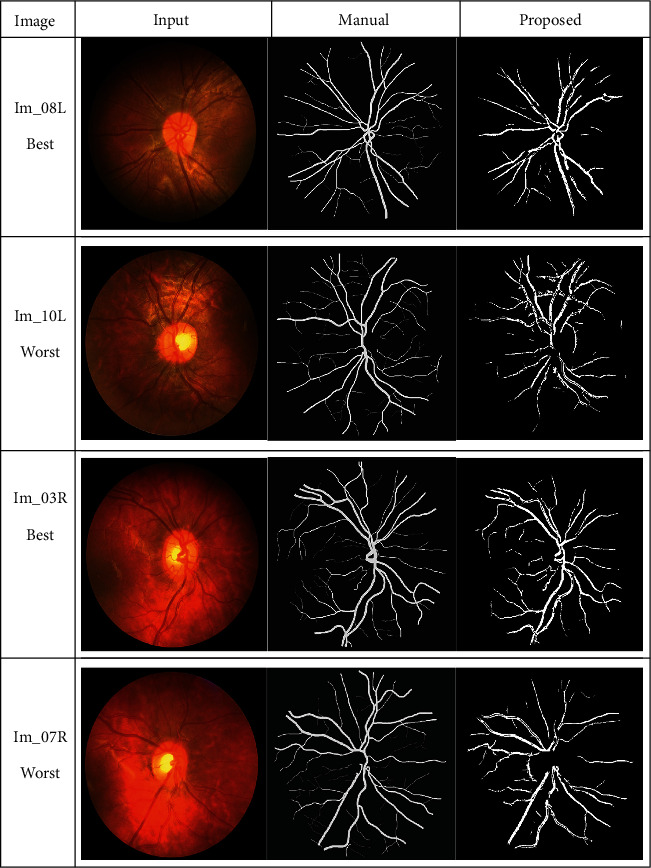
Best and worst cases visual results from the CHASE_DB1 dataset.

The success of the proposed framework is established by utilizing four freely obtainable datasets: DRIVE, STARE, HRF, and CHASE_DB1 for testing and evaluation. The average performance parameters results in Table 3 are computed by processing 20 test images of the DRIVE and STARE datasets. The performance scores of HRF dataset (15 normal images, 15 DR, and 15 glaucomatous) and CHASE_DB1 are presented in Tables 4 and 5 and Table 6, respectively. The best and worst results within Tables 3–6 are highlighted in italic font. The best and worst image results from each dataset are selected based on their accuracy's scores. Their pictorial results are shown in Figures 11–14.

The framework performs well on both healthy and pathological images of all selected datasets. The statistical results in Tables 3–6 validates that the suggested system is robust and has the capability to handle the bright lesions images of the STARE dataset, higher resolution images of the HRF dataset, low resolution images of the DRIVE dataset, and left/right eyes images of the CHASE_DB1 dataset. The anatomical structures are also efficiently omitted to avoid any misclassification.

The average statistical results of the proposed framework on all selected datasets are displayed in Table 7, which reflects that the highest mean score of Acc 0.997, Sn 0.814, Sp 0.997, and AUC 0.905 is achieved on the CHASE_DB1 dataset. The lowest FPR is also observed using the same dataset. The highest value of MCC 0.761 and CAL 0.699 is recorded on the HRF dataset. The highest value of each parameter is italicized in the respective column of the Table 7.

The average performance parameter scores of the proposed framework on the DRIVE and STARE datasets are compared with the existing literature in Table 8, while Table 9 shows the result comparison of the HRF and CHASE_DB1 datasets. The Acc, Sn, and Sp results of all techniques in Tables 8 and 9 are acquired from their respective published articles while the AUC result is calculated by using the formula in Table 2.

In Table 8, the obtained results of the framework are compared with 19 unsupervised and 18 supervised existing techniques. The proposed framework achieved the highest Acc result than all unsupervised methods on the DRIVE dataset except Khan et al. [40], Memari et al. [59] which is 0.003%, and Fan et al. [60] which is 0.002% better than ours. The supervised methods Ricci and Perfetti [14], Lupascu et al. [15], Wang et al. [16], Zhu et al. [18], Thangaraj et al. [22], Memari et al. [23], Khowaja et al. [26], and Fan et al. [61] show 0.001%, 0.001%, 0.019%, 0.003%, 0.003%, 0.014%, 0.017%, and 0.008% better results than the proposed method, respectively. But some of these methods are only validated on one dataset, which reflects that they are tuned for a single dataset. Some of these methods produce a very low AUC score, which is a trade-off between Sn and Sp. Moreover, supervised methods are computationally very expensive. In the case of the STARE dataset, the framework produced highest Acc scores than all other methods. Table 9 reflects that there are very few techniques that used both HRF and CHASE_DB1 datasets for validation. The Acc score of the framework is higher than both supervised and unsupervised approaches on the HRF and CHASE_DB1 datasets except Soomro et al. [62] and Fan et al. [61] which is slightly higher than ours on HRF dataset only. Fan et al. [61] showed higher Sp value than all other methods on the HRF dataset. The highest Sp value on CHASE_DB1 dataset is obtained by the proposed method. All the other supervised and unsupervised methods acquired a bit greater or equivalent values of Sn and AUC metric on the HRF and CHASE_DB1 datasets as compared to ours.

In Table 10, the MCC and CAL values are recorded by the proposed method and other existing supervised and unsupervised methods. The MCC and CAL values of Chauduri et al. [31], Niemeijer et al. [11], Hoover et al. [53], and B-COSFIRE [5] are calculated by utilizing their publicly accessible segmented images. The results of Fraz et al. [68, 69], RUSTICO [58], Yang et al. [70, 71], Vega et al. [72], FC-CRF [73], and UP-CRF [73] are extracted from their published articles.

The average value of MCC attained by the proposed method is higher than all compared unsupervised approaches on the DRIVE, STARE, and HRF datasets, while it is statistically lower than the supervised methods (i.e., FC-CRF [73] and UP-CRF [73]) on the DRIVE, STARE, and CHASE_DB1 datasets. The CAL value of the proposed method is observed higher than all supervised and unsupervised methods on the HRF dataset, while it is statistically lower than or equivalent to CAL values of other methods on the DRIVE, STARE, and CHASE_DB1 datasets.

#### 4.3.1. Processing Time

The proposed framework processes a single image in a very short time as equated to other approaches in Table 11. The time values are computed on the single image taken from the DRIVE and STARE datasets.

## 5. Conclusion

Vessel extraction is momentous for inspecting abnormalities inside and around the retinal periphery. The retinal vessel segmentation is a challenging task due to the existence of pathologies, unpredictable dimensions and contour of the vessels, nonuniform clarification, and structural inconsistency between subjects. The proposed methodology is consistent, faster, and completely automated for isolation of retinal vascular network. The success of the proposed framework is evidently revealed by the RVS statistics on the DRIVE, STARE, HRF, and CHASE_DB1 datasets. The eradication of anomalous structures prior to enhancement boosted the efficiency of the proposed method. The application of logical operators avoids misclassification of foreground pixels which enhances the accuracy and makes the method robust. Pictorial representation validates that the framework is able to segment both healthy and unhealthy images. Furthermore, the method does not include any hand-marked data by experts for training, which makes it computationally fast.

## Figures and Tables

**Algorithm 1:**
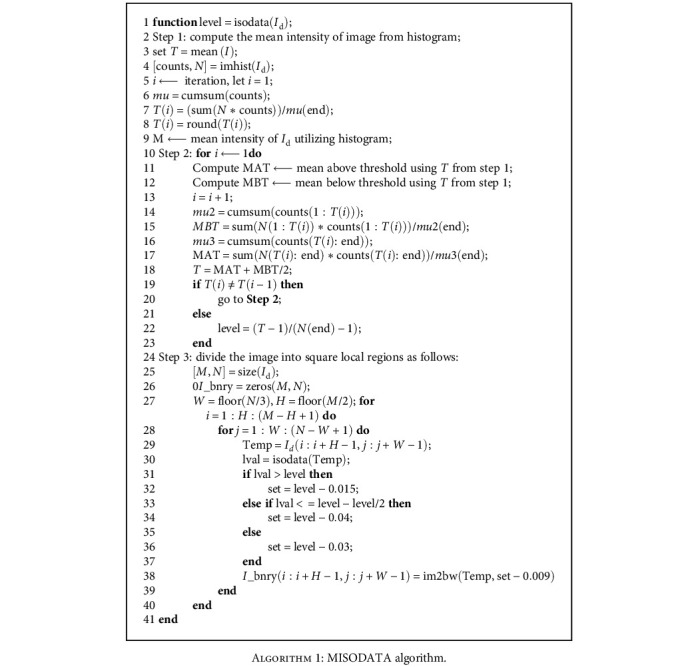
MISODATA algorithm.

**Table 1:** Datasets comparison.

Dataset	Image classification	Image size	Format
DRIVE	Total 40 images	565 × 584	JPEG
	20 test, 20 training		
	7 abnormal, 33 normal		

STARE	Total 20 images	700 × 605	PPM
	10 normal		
	10 abnormal		

HRF	Total 45 images	3504 × 2336	JPEG
	15 normal, 15 DR		
	15 glaucomatous		

CHASE_DBI	Total 28 images	1280 × 960	JPEG
	14 left eye		
	14 right eye		

**Table 2:** Performance judgment criteria of the proposed model.

Parameter	Formulation
Sensitivity (Sn)	$\frac{\text{TP}}{\text{TP}+\text{FN}}$
Specificity (Sp)	1-FPR or TN/TN + FP
Accuracy (Acc)	$\frac{\text{TN}+\text{TP}}{\text{TN}+\text{FP}+\text{TP}+\text{FN}}$
Area under ROC curve (AUC)	Sn + Sp/2
Matthews correlation coefficient (MCC)	$\frac{\text{TP}/N-S\times P}{\sqrt{P\times S\times \left(1-S\right)\times }\left(1-P\right)}$
Connectivity-area-length (CAL)	*f*(*C*, *A*, *L*) = *C* × *A* × *L*

**Table 3:** Statistical scores achieved on the DRIVE and STARE datasets.

Img	DRIVE						STARE					
	Acc	Sn	Sep	AUC	MCC	CAL	Acc	Sn	Sep	AUC	MCC	CAL
1	0.961	0.810	0.976	0.893	0.764	0.764	0.993	0.494	0.997	0.745	0.468	0.550
2	0.966	0.771	*0.988*	0.883	*0.802*	*0.783*	0.996	0.832	0.977	0.915	0.594	0.418
3	0.948	0.812	0.961	0.887	0.712	0.660	*0.988*	0.838	*0.988*	0.913	0.476	0.539
4	0.967	0.757	0.987	0.872	0.786	0.701	0.995	*0.419*	0.999	*0.709*	*0.383*	*0.254*
5	*0.967*	0.792	0.983	0.888	0.780	0.767	0.989	0.909	0.989	0.949	0.625	0.575
6	0.957	0.755	0.978	0.866	0.741	0.653	0.995	0.929	0.996	0.962	0.735	0.663
7	0.959	0.821	0.969	0.895	0.721	0.657	0.996	0.785	0.997	0.891	0.709	*0.684*
8	0.947	0.843	*0.954*	0.899	*0.660*	0.621	0.997	0.792	0.998	0.895	0.714	0.678
9	0.955	0.771	0.971	0.871	0.709	0.632	0.998	0.828	0.999	0.914	0.843	0.655
10	0.962	0.819	0.973	0.896	0.736	0.731	0.993	0.941	0.993	0.967	0.695	0.576
11	0.958	0.795	0.973	0.884	0.737	0.681	0.999	0.971	0.999	0.985	0.892	0.617
12	0.955	0.856	0.964	0.910	0.739	0.750	*0.999*	0.954	*0.999*	0.977	*0.961*	0.677
13	0.955	*0.703*	0.983	*0.843*	0.736	0.672	0.998	0.842	0.999	0.921	0.819	0.551
14	0.952	*0.869*	0.959	*0.914*	0.717	0.689	0.997	0.867	0.999	0.933	0.848	0.613
15	*0.947*	0.783	0.960	0.872	0.666	*0.547*	0.998	0.762	0.999	0.881	0.773	0.685
16	0.962	0.798	0.977	0.888	0.762	0.743	0.995	0.659	0.998	0.829	0.714	0.489
17	0.954	0.836	0.963	0.899	0.716	0.698	0.999	*0.975*	0.999	*0.987*	0.961	0.612
18	0.961	0.813	0.976	0.895	0.771	0.730	0.999	0.730	0.999	0.865	0.829	0.531
19	0.961	0.754	0.984	0.869	0.775	0.709	0.999	0.764	0.999	0.882	0.538	0.550
20	0.957	0.773	0.976	0.875	0.746	0.722	0.996	0.547	0.998	0.773	0.562	0.397
Avg	*0.958*	*0.797*	*0.973*	*0.885*	*0.739*	*0.696*	*0.996*	*0.792*	*0.997*	*0.895*	*0.707*	*0.566*

**Table 4:** Average efficiency scores on the HRF dataset (normal and diabetic images).

Img	Normal						Diabetic					
	Acc	Sn	Sep	AUC	MCC	CAL	Acc	Sn	Sep	AUC	MCC	CAL
1	0.958	*0.701*	0.988	0.844	0.700	0.574	0.963	0.768	0.977	0.845	0.646	*0.742*
2	0.965	0.806	0.983	0.894	0.787	0.671	0.958	0.755	0.974	0.843	0.671	0.672
3	*0.954*	0.713	0.983	0.848	0.741	0.632	0.949	0.775	0.961	*0.860*	0.631	0.563
4	0.957	0.810	*0.975*	*0.882*	0.761	0.719	0.951	0.758	0.966	0.836	0.633	0.531
5	0.968	0.765	0.988	0.876	0.795	0.754	*0.964*	0.725	0.983	0.823	0.709	0.609
6	0.960	0.832	0.978	0.888	0.774	0.759	0.950	*0.564*	*0.988*	*0.728*	*0.619*	*0.394*
7	0.968	0.817	0.988	0.878	0.793	0.741	0.957	0.737	0.980	0.834	0.702	0.688
8	0.964	0.806	0.985	0.879	0.788	0.729	0.952	*0.776*	0.971	0.841	0.680	0.688
9	0.956	0.808	0.968	0.888	*0.681*	0.632	0.945	0.758	*0.958*	0.858	0.634	0.551
10	0.959	0.765	0.979	0.863	0.730	*0.561*	0.951	0.664	0.981	0.809	0.684	0.598
11	*0.970*	0.839	*0.990*	0.886	0.806	0.753	0.953	0.700	0.983	0.808	*0.713*	0.611
12	0.967	*0.852*	0.986	0.895	*0.816*	*0.788*	0.955	0.701	0.976	0.813	0.666	0.579
13	0.966	0.837	0.982	0.894	0.778	0.711	0.960	0.753	0.981	0.829	0.704	0.645
14	0.966	0.835	0.981	0.890	0.761	0.699	*0.944*	0.726	0.964	0.829	0.645	0.622
15	0.970	0.827	0.982	*0.905*	0.708	0.768	0.953	0.730	0.971	0.834	0.648	0.547
Avg	0.963	0.801	0.982	0.881	0.761	0.699	0.954	0.726	0.974	0.826	0.666	0.603

**Table 5:** Average efficiency scores on the HRF dataset (Glaucomatous images).

Img	Glaucomatous					
	Acc	Sn	Sep	AUC	MCC	CAL
1	0.952	0.813	*0.962*	0.887	0.688	*0.743*
2	0.954	0.805	0.966	0.886	0.704	0.679
3	0.962	0.830	0.970	0.900	0.688	*0.610*
4	0.962	0.788	0.974	0.881	0.711	0.695
5	0.963	*0.833*	0.972	*0.902*	*0.721*	0.663
6	0.962	0.811	0.973	0.892	0.708	0.713
7	*0.969*	0.773	*0.977*	0.875	0.721	0.609
8	0.957	0.835	0.964	0.899	0.703	0.720
9	0.962	0.774	0.975	0.875	0.710	0.646
10	0.963	0.780	0.976	0.878	0.718	0.665
11	0.962	0.749	0.976	0.863	0.717	0.641
12	0.958	0.799	0.967	0.883	0.717	0.688
13	0.958	0.753	0.974	0.863	0.692	0.664
14	*0.951*	0.764	0.965	0.865	*0.663*	0.646
15	0.956	*0.748*	0.973	*0.860*	0.691	0.620
Avg	0.959	0.790	0.979	0.881	0.703	0.667

**Table 6:** Average performance achieved on the CHASE_DB1 dataset.

Images	Acc	Sn	Sep	AUC	MCC	CAL
01L	0.995	0.753	0.996	0.874	0.582	0.552
01R	0.997	0.705	*0.999*	0.852	0.707	0.485
02L	*0.995*	0.647	0.997	0.822	0.623	0.516
02R	0.995	0.744	0.996	0.870	0.620	0.488
03L	0.996	0.783	0.997	0.890	0.646	0.571
03R	0.998	0.848	0.999	0.923	*0.793*	0.582
04L	0.996	*0.588*	0.998	*0.793*	0.648	0.616
04R	0.997	0.651	0.998	0.825	0.692	0.535
05R	0.997	0.758	0.998	0.878	0.746	0.634
06L	0.998	0.620	0.999	0.809	0.694	0.548
06R	0.998	0.705	0.999	0.852	0.726	0.503
07L	0.997	0.722	0.998	0.860	0.678	0.574
07R	0.997	0.630	0.999	0.815	0.675	0.553
08L	0.997	0.850	0.998	0.924	0.650	0.507
08R	0.997	0.867	0.997	0.932	0.641	0.566
09L	0.991	0.816	*0.991*	0.903	*0.315*	0.509
09R	0.994	0.915	0.994	0.955	0.368	0.493
10L	0.994	0.672	0.995	0.834	0.408	0.483
10R	0.996	0.900	0.996	0.948	0.590	0.458
11L	0.997	*0.933*	0.997	*0.965*	0.617	0.622
11R	*0.998*	0.878	0.998	0.938	0.656	0.570
12L	0.997	0.699	0.998	0.848	0.566	0.609
12R	0.998	0.770	0.999	0.885	0.693	0.513
13L	0.998	0.833	0.998	0.916	0.667	0.503
13R	0.998	0.650	0.998	0.824	0.548	*0.445*
14L	0.997	0.813	0.998	0.905	0.652	*0.639*
14R	0.997	0.692	0.999	0.845	0.628	0.554
Average	*0.997*	*0.757*	*0.997*	*0.877*	*0.629*	*0.547*

**Table 7:** Mean results comparison of the proposed method on different datasets.

Datasets	Images	Acc	Sn	Sp	AUC	MCC	CAL
DRIVE (1^st^ observer)	20	0.954	0.766	0.972	0.869	0.721	0.690
DRIVE (2^nd^ observer)		0.958	0.797	0.973	0.885	0.739	0.696
HRF_Normal	15	0.963	0.801	0.982	0.881	*0.761*	*0.699*
HRF_DR		0.954	0.726	0.974	0.826	0.666	0.603
HRF_Glaucoma		0.959	0.790	0.979	0.881	0.703	0.667
HRF_Average	45	0.959	0.772	0.978	0.863	0.710	0.656
CHASE_DB1 (1^st^ observer)	28	*0.997*	0.757	*0.97*	0.877	0.629	0.547
CHASE_DB1 (2^nd^ observer)		0.996	*0.814*	0.996	*0.905*	0.569	0.547
STARE	20	0.996	0.792	0.997	0.895	0.707	0.566

**Table 8:** Performance assessments of existing techniques on the DRIVE and STARE datasets.

Method	Year	DRIVE				STARE			
		Acc	Sn	Sp	AUC	Acc	Sn	Sp	AUC
Human observer		0.947	0.779	0.972	0.874	0.935	0.895	0.938	0.917
Unsupervised techniques									
Chauduri [31]	1989	0.877	—	—	0.788	—	—	—	—
Zana and Klein [42]	2001	0.938	0.697	—	—	—	—	—	—
Martinez-Perez [45]	2007	0.934	0.725	0.965	0.845	0.941	0.751	0.955	0.853
Zhang [27]	2010	0.938	—	—	—	0.948	—	—	—
Bankhead [38]	2012	0.937	0.703	0.971	0.837	0.932	0.758	0.950	0.854
Fraz [44]	2012	0.943	0.715	0.976	0.845	0.944	0.731	0.968	0.850
Azzopardi [5]	2015	0.944	0.766	0.970	0.868	0.950	0.772	0.970	0.871
Oliveira [33]	2016	0.946	0.864	0.956	0.910	0.953	0.825	0.965	0.895
Khan [40]	2016	0.961	0.746	0.980	0.863	0.946	0.758	0.963	0.861
Biswal [29]	2017	0.950	0.710	0.970	0.840	0.950	0.700	0.970	0.835
Khan [32]	2017	0.944	0.754	0.964	0.859	0.948	0.752	0.956	0.854
Soomro [63]	2017	0.943	0.752	0.976	0.864	0.961	0.784	0.981	0.883
Badawi [3]	2018	0.955	0.791	0.971	0.881	0.953	0.865	0.961	0.913
Yue [50]	2018	0.945	0.753	0.973	0.863	—	—	—	—
Soomro [9]	2018	0.948	0.745	0.962	0.854	0.951	0.784	0.976	0.880
Soomro [64]	2018	0.953	0.752	0.976	0.864	0.967	0.786	0.982	0.884
Fan [60]	2018	0.960	0.736	0.981	0.858	0.957	0.791	0.970	0.880
Khan [65]	2019	0.951	0.770	0.965	0.868	0.951	0.752	0.981	0.867
Memari [59]	2019	0.961	0.761	0.981	0.871	0.951	0.782	0.965	0.873
Proposed	*2020*	*0.958*	*0.797*	*0.973*	*0.885*	*0.996*	*0.792*	*0.998*	*0.895*
Supervised techniques									
Niemeijer [11]	2004	0.942	0.690	0.970	0.830	—	—	—	—
Staal [12]	2004	0.944	0.719	0.977	0.848	0.952	0.697	0.981	0.839
Ricci [14]	2007	0.959	—	—	—	0.964	—	—	—
Lupascu [15]	2010	0.959	0.673	0.987	0.830	—	—	—	—
MarÃ­n [13]	2011	0.945	0.707	0.980	0.844	0.953	0.694	0.982	0.838
Wang [16]	2015	0.977	0.817	0.973	0.895	0.981	0.810	0.979	0.894
Roychowdhury [17]	2015	0.952	0.725	0.983	0.854	0.951	0.772	0.973	0.873
Aslani [20]	2016	0.951	0.754	0.980	0.867	0.961	0.755	0.983	0.869
Zhu [18]	2017	0.961	0.714	0.987	0.851	—	—	—	—
Thangaraj [22]	2017	0.961	0.801	0.975	0.888	0.943	0.834	0.954	0.893
Memari [23]	2017	0.972	0.872	0.988	0.930	0.951	0.809	0.979	0.894
Dharmawan [21]	2018	—	0.831	0.972	0.902	—	0.792	0.983	0.887
Yan [24]	2018	0.954	0.763	0.982	0.873	0.964	0.774	0.986	0.880
Guo [25]	2019	0.955	0.780	0.981	0.881	0.966	0.820	0.983	0.902
Khowaja [26]	2019	0.975	0.818	0.971	0.895	0.975	0.824	0.975	0.899
Soomro [8]	2019	0.959	0.802	0.974	0.948	0.961	0.801	0.969	0.945
Soomro [62]	2019	0.956	0.870	0.985	0.986	0.968	0.848	0.986	0.988
Fan [61]	2019	0.966	0.796	0.982	0.889	0.974	0.816	0.987	0.901

**Table 9:** Performance assessments of existing methods with the proposed model on the HRF and CHASE_DB1 datasets.

Technique	Year	HRF				CHASE_DB1			
		Acc	Sn	Sp	AUC	Acc	Sn	Sp	AUC
Unsupervised techniques									
Odstrcilik [54]	2013	0.949	0.774	0.967	0.871	—	—	—	—
Azzopardi [5]	2015	—	—	—	—	0.939	0.759	0.959	0.859
Zhang [66]	2016	0.957	0.798	0.974	0.886	0.946	0.763	0.968	0.866
Biswal [29]	2017	—	—	—	—	0.940	0.760	0.970	0.865
Rodrigues [67]	2017	0.948	0.722	0.964	0.843	—	—	—	—
Badawi [3]	2018	—	—	—	—	0.953	0.800	0.964	0.882
Proposed	*2020*	0.960	0.732	0.979	0.855	0.996	0.727	0.997	0.862
Supervised techniques									
Roychowdhury [17]	2015	—	—	—	—	0.953	0.720	0.982	0.851
Thangaraj [22]	2017	—	—	—	—	0.947	0.629	0.973	0.797
Memari [23]	2017	—	—	—	—	0.948	0.819	0.959	0.889
Dharmawan [21]	2018	—	0.813	0.977	0.895	—	—	—	—
Yan [24]	2018	—	—	—	—	0.961	0.764	0.981	0.873
Fan [60]	2018	—	—	—	—	0.951	0.657	0.973	0.815
Guo [25]	2019	—	—	—	—	0.963	0.789	0.980	0.885
Khowaja [26]	2019	—	—	—	—	0.952	0.756	0.976	0.866
Soomro [62]	2019	0.962	0.829	0.962	0.978	0.976	0.886	0.982	0.985
Fan [61]	2019	0.976	0.824	0.987	0.905	0.971	0.802	0.985	0.893

**Table 10:** Performance assessments of existing techniques on the four datasets.

Method	Year	DRIVE		STARE		HRF		CHASE_DB1	
		MCC	CAL	MCC	CAL	MCC	CAL	MCC	CAL
Unsupervised techniques									
Chauduri [31]	1989	0.420	0.208	—	—	—	—	—	—
Hoover [53]	2000	—	—	0.615	0.534	—	—	—	—
Fraz [68]	2011	0.733	—	0.700	—	—	—	—	—
Fraz [69]	2013	0.736	—	0.691	—	—	—	—	—
B-COSFIRE [5]	2015	0.719	0.721	0.698	0.709	0.686	0.577	0.656	0.608
RUSTICO [58]	2019	0.729	0.728	0.698	0.709	0.691	0.587	0.663	0.620
Proposed	2020	0.739	0.696	0.707	0.566	0.710	0.656	0.629	0.547
Supervised techniques									
Yang [70]	2019	0.736	—	0.704	—	0.712	—	—	—
Yang [71]	2018	0.725	—	0.662	—	0.682	—	—	—
FC-CRF [73]	2016	0.756	0.731	0.727	0.658	0.690	0.541	0.704	0.622
UP-CRF [73]	2016	0.740	0.675	0.726	0.665	0.677	0.475	0.689	0.571
Vega [72]	2015	0.662	—	0.640	—	—	—	—	—
Niemeijer [11]	2004	0.722	0.659	—	—	—	—	—	—

**Table 11:** Processing time evaluation of the systems.

Method	Time	Hardware particulars
Roychowdhury [17]	3.11 sec	Intel Core i3 CPU 2.6 GHz, 2 GB RAM
Zhu [18]	12.160 sec	4.0 GHz Intel i7-4790K CPU and 32 GB RAM
Memari [23]	8.2 mins	Intel i5-M480 CPU, 2.67 GHz, 4 GB RAM
Biswal [29]	3.3 sec	Intel i3 (4010U CPU) 1.7 GHz, 4 GB RAM
Badawi [3]	8 sec	CPU 2.7 GHz, 16 GB RAM
Yue [50]	4.6 sec	Intel i5-6200U CPU 2.3 GHz, 8 GB RAM
Khan [39]	6.1 sec	5∗Intel Core i3 CPU, 2.53 GHz, 4 GB RAM
Khan [40]	1.56 sec	
Azzopardi [5]	11.83 sec	
Vlachos [47]	9.3 sec	
Bankhead [38]	22.45 sec	
Proposed	5.5 sec	

## Data Availability

All the data are fully available within the manuscript without any restriction.
